# Retraction: Antiviral effects of novel herbal medicine KIOM-C, on diverse viruses

**DOI:** 10.1371/journal.pone.0355659

**Published:** 2026-08-11

**Authors:** The PLOS One Editors

Following the publication of this article [1], concerns were raised regarding results presented in Figs 1 and 2, the statistical methodology, and undisclosed competing interests. Specifically,

- The following panels appear similar:
  - Fig 2B p65 in [1] and Fig 4C TBK1 in [2].
  - Fig 2B ERK in [1] and Fig 4C ERK in [2] despite being used to represent different experimental conditions.
- The Statistical Analysis subsection of the Materials and Methods section reports that statistical analyses were carried out using Student’s *t*-test, however multiple experiments in [1] appear to present more than two groups and/or multiple variables.
- The Competing interests statement states that the authors have declared that no competing interests exist, despite corresponding author JYM being listed as an inventor on patent PCT/KR2010/000107 [3] which is linked to the medicine KIOM-C tested in this study.

Regarding the image concerns, corresponding author JSL stated that the studies reported in [1] and [2] were run in parallel and the original blot data for these studies were inadvertently saved in the same folder. They stated that the original image data and individual-level data underlying the results in Figs 1, 2, and 4E are no longer available, but provided the individual-level data underlying Table 3 and the graphs presented in Figs 3B, 4A-D, S1A, and S1B. Editorial assessment of these data raised additional concerns for these data that call into question the reliability of the reported results.

Regarding the statistical concerns, corresponding author JSL stated that the comparisons strictly used Student’s *t*-tests to evaluate the differences between the untreated control group and the KIOM-C treated experimental groups, and that neither pairwise multi-comparison adjustment nor multivariate analysis was not required for this study. Contrary to this explanation, the figure legend for Fig 4 states that comparisons of groups were analyzed by Student *t*-tests and ANOVA. PLOS remains concerned about the statistical methodology used for the data analysis reported in this study.

Regarding the undisclosed competing interest, corresponding author JSL stated that the patent number was reported in the article, but was omitted from the competing interests declaration due to an administrative oversight.

In light of the unresolved concerns, which call into question the reliability and integrity of the published results, the *PLOS One* Editors retract this article.

MRT, PW, THK, WKC, CJK, JYM, and JSL did not agree with the retraction. MYEC and MEP either did not respond directly or could not be reached. JSL stands by the scientific validity and reproducibility of the findings.
